# Pharmacokinetic Modeling of the “Nose-to-Brain” Pathway as Demonstrated by Intranasal Administration of Cannabidiol-Loaded Nanoparticles

**DOI:** 10.3390/ph19030456

**Published:** 2026-03-11

**Authors:** Ilya Eydelman, Shimon Ben-Shabat, Amnon C. Sintov

**Affiliations:** 1 Department of Clinical Biochemistry and Pharmacology, Ben-Gurion University of the Negev, P.O. Box 653, Be’er Sheva 8410501, Israel; eydelman@bgu.ac.il; 2 Laboratory for Biopharmaceutics, E.D. Bergmann Campus, Ben-Gurion University of the Negev, P.O. Box 653, Be’er Sheva 8410501, Israel; 3 Department of Biomedical Engineering, Ben Gurion University of the Negev, P.O. Box 653, Be’er Sheva 8410501, Israel

**Keywords:** pharmacokinetic model, intranasal delivery, brain drug levels, polymeric nanoparticles, cannabidiol

## Abstract

**Background/Objectives**: Cannabidiol is a non-psychoactive substance that possesses properties suitable for the treatment of several disorders related to the central nervous system. However, successful administration of cannabidiol remains challenging due to low and variable bioavailability and potential adverse effects. Intranasal delivery of cannabidiol may help overcome these limitations, but the pharmacokinetics of such administration has not been fully established. **Methods**: Starch-based cannabidiol-loaded nanoparticles were used as carriers and were administered to rats via the intranasal route. Cannabidiol levels in plasma and the brain were examined at different time points and compared to cannabidiol levels in plasma and the brain following intravenous administration of cannabidiol solution for injection. Pharmacokinetic parameters were calculated for each delivery route, and a pharmacokinetic model was fitted for the intranasal administration. **Results**: Intranasal administration resulted in a bioavailability of 47.9%. Systemic absorption accounted for 44% of the absorbed drug, while 56% was absorbed by direct brain entry. Intranasal administration resulted in rapid brain penetration with a *brain t*_max_ of 10 min and demonstrated a brain bioavailability of 28.5% compared to bioavailability after intravenous bolus injection of cannabidiol solution. **Conclusions**: Intranasal administration of cannabidiol-loaded nanoparticles was found to be effective for the delivery of cannabidiol to the brain with significantly lower systemic exposure compared to intravenous administration. A proposed pharmacokinetic model was found to be appropriate in describing and predicting the disposition pathways following intranasal administration, especially when designing drug delivery systems for brain targeting.

## 1. Introduction

Cannabidiol (CBD) is one of more than a hundred phytocannabinoid compounds in *Cannabis sativa*. As a non-psychoactive molecule, CBD has a good safety profile and displays several important protective effects, such as anti-inflammatory, anti-oxidative and anti-necrotic [1,2,3,4]. Consequently, CBD appears suitable for the treatment of several central nervous system conditions such as epilepsy, Alzheimer’s disease, Parkinson’s disease and multiple sclerosis [1,5]. Indeed, the activity of CBD has been reported in several pathology models as well as in clinical settings with several CBD-containing formulations that have been approved for human use [4,6]. Recently, the use of CBD was reported to increase liver enzymes and drug-induced liver injury [7]. Although these events were observed at daily doses far exceeding those commonly used [7,8], and despite numerous reports describing the beneficial hepatic effects of CBD [9,10], such findings have nonetheless heightened awareness of the need to minimize unintended systemic CBD exposure.

Despite the therapeutic potential of CBD, the successful delivery of CBD remains a challenge. Following oral administration, which is the most convenient and patient-compliant, CBD bioavailability is relatively low and variable: 6–19% [4,5,11] due to irregular absorption and extensive hepatic metabolism (first-pass metabolism). Xu et al. [12] reported an approximate oral bioavailability of only 8.6% (based on a period of 24 h) after oral administration of 20 mg/kg CBD to mice. Thus, a more effective alternative route of administration must be sought.

Intranasal (IN) drug delivery is a non-invasive and safe method that offers significant advantages in the treatment of neurological and other central nervous system (CNS) diseases. Intranasal drug delivery has been demonstrated to circumvent the blood–brain barrier (BBB), by rapidly targeting the central nervous system (CNS) through the olfactory and trigeminal nerves located in the nasal cavity [13]. IN administration allows for the rapid onset of action, reduced systemic (and hepatic) side effects compared to the oral and parenteral routes, enhanced brain targeting, and improved patient compliance [14]. However, optimizing drug dosage forms and devices that are specifically designed for “nose-to-brain” drug delivery remains crucial to ensure consistent dosing and therapeutic outcomes. Such optimization is naturally based on the comparison of the pharmacokinetics (brain and plasma) of the drug administered via the IN route with its intravenous (IV) administration. The data obtained from the intravenous administration of a drug is required for calculating pharmacokinetic parameters such as the elimination rate constant and the absolute bioavailability. Thus far, the pharmacokinetics of CBD based on IN administration has not been established, while only a few reports have compared CBD administrations via these two routes. To date, explicit compartmental models for CBD intranasal pharmacokinetics in rodents have not been widely published. Existing reports mainly employed non-compartmental analysis [15,16], a one-compartment model [17], non-specified calculation of pharmacokinetic parameters [18] or a descriptive regional distribution analysis [19]. Ahmed et al. [18] reported that the brain/blood ratios of CBD after 30 min following IV and IN administrations of the drug to rats (3.1 mg doses suspended in 0.5 mL or 0.1 mL saline) were found to be 0.20 ± 0.25 and 0.61 ± 0.16, respectively, and CBD bioavailability (F%) was 48.5%. Following this high dose of CBD, the peak brain concentrations (*brain C*_max_) were 2578.12 ± 26.1 ng/mL (IV) and 3310.41 ± 32.8 ng/mL (IN) after 2 h (brain *t*_max_) [18]. Wang et al. [16] analyzed the pharmacokinetics of CBD formulated in nanostructured lipid carriers (NLCs) and intranasally administered to rats. The IN administration (300 µg in a 400 mL volume) was compared to the IV administration of CBD solution at the same dosage. The researchers found that the peak plasma concentrations after both administrations were similar (IN-175.03 ± 2.71 ng/mL and IV-173.45 ± 24.80 ng/mL), and brain concentrations (*brain C*_max_) were 141.31 ± 51.34 ng/mL (IV) and 153.38 ± 33.51 ng/mL (IN) [16]. Drug bioavailability after intranasal CBD-NLC was 76.2%, while the brain bioavailability of the intranasal CBD-NLC increased to approximately two-fold relative to IV administration [16]. In another report, Upadhyay et al. [19] used a nano-formulation based on W/O emulsion and b-cyclodextrin in mice. They showed that 15.94% of the total intranasally administered CBD dose (182 mg/kg) was detected in the plasma, while only 0.12% reached the brain. In a previous paper published by our group [20], we presented a new CBD-loaded nano-formulation based on biodegradable crosslinked starch (SNPs). In the present paper, the pharmacokinetics of intranasal CBD is reported and demonstrated using SNPs as carriers and compared to the intravenous bolus administration of CBD solution. In addition, a specific mammillary compartmental model has been established for the intranasal delivery of drugs, in which the brain is represented as the target organ.

### Theoretical Section

Pharmacokinetic model for intranasal drug administration which includes the nose-to-brain pathway.

**Figure 1:**
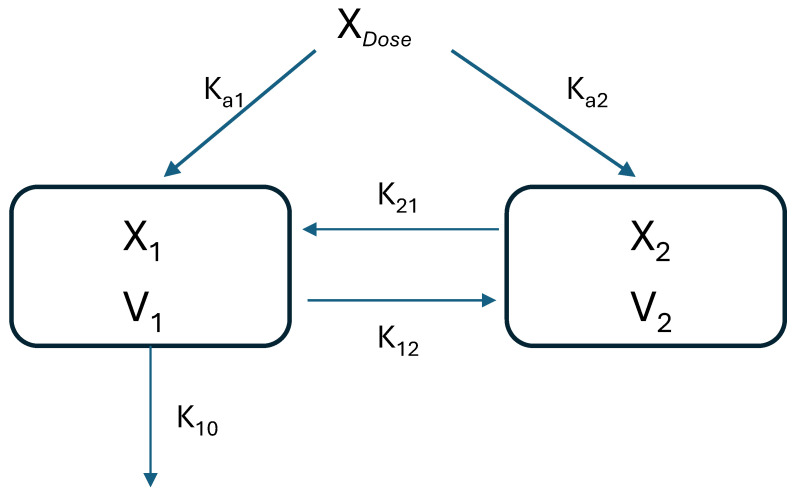
Schematic representation of drug disposition after intranasal administration, as a two-compartment open model with two routes of absorption, one to the systemic circulation (central compartment) and the other to the brain directly. The model is based on the following assumptions: first-order kinetics, no drug elimination from the brain, and an immediate or very rapid drug release/dissolution. Note: The pharmacokinetic model is based on the administration of a low drug dosage; thus, the pharmacokinetics of CBD at doses of 0.3–0.4 mg/kg body weight (as presented in this paper) can be adequately described by first-order or linear processes.

X_Dose_ (Figure 1) is the drug quantity absorbed into the body, which is the fraction F (bioavailability) of the dose administered intranasally. X_1_ and X_2_ are the amounts of drug in the central compartment (systemic circulation and elimination organs) and the brain, respectively. V_1_ and V_2_ are the volumes of distribution in the central compartment and the brain, respectively. K_12_ and K_21_ are the first-order rate constants of distribution and K_10_ is the first-order process of metabolism and excretion (elimination rate constant). It has been well-established, including in the data presented in this paper, that CBD undergoes an extensive liver metabolism; therefore, it is a high-level assumption that the main route of elimination of CBD occurs from the central compartment (K_10_) rather than from the peripheral tissue. Ka_1_ and Ka_2_ are the first-order absorption rate constants into the central compartment and the brain, respectively. It is to be noted that, although this model is specific for the brain compartment, it also includes drugs that display multi-compartment characteristics after intravenous injection. However, following extravascular administration, i.e., via the intranasal route, we often fail to observe a distributive phase even though the overall distribution rate constants exist.

The rate of drug absorption into the two compartments is described as follows:

(1) $$\frac{dX_{Dose}}{dt}=-K_{a1}X_{Dose}-K_{a2}X_{Dose}$$

The Laplace transform of the absorbed amount of drug according to the proposed compartmental model is given below:

(2) $$S{\bar{X}}_{Dose}-F\cdot Dose=-K_{a1}{\bar{X}}_{Dose}-K_{a2}{\bar{X}}_{Dose}$$

(3) $${\bar{X}}_{Dose}\left(S+K_{a1}+K_{a2}\right)=F\cdot Dose$$

(4) $${\bar{X}}_{Dose}=\frac{F\cdot Dose}{\left(S+K_{a1}+K_{a2}\right)}$$

where S is the standard notation used in Laplace operations.

The amount of drug present in the brain compartment as a function of time is given in the following equations:

(5) $$\frac{dX_{2}}{dt}=-K_{21}X_{2}+K_{12}X_{1}+K_{a2}X_{Dose}$$

Using Laplace transform for Equation (5) combined with Equation (4):

(6) $$S{\bar{X}}_{2}-0=-K_{21}{\bar{X}}_{2}+K_{12}{\bar{X}}_{1}+\frac{K_{a2}\cdot F\cdot Dose}{\left(S+K_{a1}+K_{a2}\right)}$$

As Ka_1_ + Ka_2_ = Ka (Ka is the overall rate constant of absorption), Equation (6) can be written as follows:

(7) $$S{\bar{X}}_{2}-0=-K_{21}{\bar{X}}_{2}+K_{12}{\bar{X}}_{1}+\frac{K_{a2}\cdot F\cdot Dose}{\left(S+K_{a}\right)}$$

or:

(8) $${\bar{X}}_{2}=\frac{{(K}_{12}{\bar{X}}_{1})\left(S+K_{a}\right)+K_{a2}\cdot F\cdot Dose}{(S+K_{21})\left(S+K_{a}\right)}$$

The amount of drug present in the central compartment as a function of time is given in the following equations:

(9) $$\frac{dX_{1}}{dt}=-K_{12}X_{1}+K_{21}X_{2}+K_{a1}X_{Dose}-K_{10}X_{1}$$

Using Laplace transform for Equation (9) combined with Equations (4) and (8):

(10) $$S{\bar{X}}_{1}-0=-K_{12}{\bar{X}}_{1}+K_{21}{\bar{X}}_{2}-K_{10}{\bar{X}}_{1}+\frac{K_{a1}\cdot F\cdot Dose}{\left(S+K_{a}\right)}$$

(11) $$S{\bar{X}}_{1}-0=-K_{12}{\bar{X}}_{1}+K_{21}\frac{{(K}_{12}{\bar{X}}_{1})\left(S+K_{a}\right)+K_{a2}\cdot F\cdot Dose}{(S+K_{21})\left(S+K_{a}\right)}-K_{10}{\bar{X}}_{1}\;+\frac{K_{a1}\cdot F\cdot Dose}{\left(S+K_{a}\right)}$$

After reorganizing:

(12) $${\bar{X}}_{1}=\frac{F\cdot Dose{\cdot [K_{a1}\left(S+K_{21}\right)+(K}_{21}K_{a2})]}{\left(S+K_{a}\right)[\left(S+K_{21}\right)\left(S+K_{10}+K_{12}\right)-K_{21}K_{12}]}$$

λ_1_ and λ_2_ are the hybrid rate constants reflecting all distribution rate processes. They are defined as {λ_1_ + λ_2_ = K_12_ + K_21_ + K_10_} and {λ_1_·λ_2_ = K_21_·K_10_}. Thus, the Laplace transform of the drug disposition in the central compartment (Equation (12)) can be given by the following:

(13) $${\bar{X}}_{1}=\frac{F\cdot Dose{\cdot [K_{a1}\left(S+K_{21}\right)+(K}_{21}K_{a2})]}{\left(S+K_{a}\right)\left(S+\lambda_{1}\right)\left(S+\lambda_{2}\right)}$$

By taking the “inverse transform” of Equation (13) using the usual method [21], Equation (14) is obtained:

(14) $$X_{1}=F\cdot Dose\cdot [\frac{{[K_{a1}\left(K_{21}-K_{a}\right)+(K}_{21}K_{a2})]}{\left(\lambda_{1}-K_{a}\right)\left(\lambda_{2}-K_{a}\right)}e^{-K_{a}t}\;+\frac{{[K_{a1}\left(K_{21}-\lambda_{1}\right)+(K}_{21}K_{a2})]}{\left(K_{a}-\lambda_{1}\right)\left(\lambda_{2}-\lambda_{1}\right)}e^{-\lambda_{1}t}\;+\frac{{[K_{a1}\left(K_{21}-\lambda_{2}\right)+(K}_{21}K_{a2})]}{\left(K_{a}-\lambda_{2}\right)\left(\lambda_{1}-\lambda_{2}\right)}e^{-\lambda_{2}t}]$$

or:

$$A_{1}e^{-K_{a}t}+A_{2}e^{-\lambda_{1}t}+A_{3}e^{-\lambda_{2}t}\;\;\;\;\left(K_{a}\;>\;\lambda_{1}\;>\;\lambda_{2}\right)$$

(15) $$C_{1}=\frac{F\cdot Dose}{V_{1}}\cdot [\frac{{[K_{a1}\left(K_{21}-K_{a}\right)+(K}_{21}K_{a2})]}{\left(\lambda_{1}-K_{a}\right)\left(\lambda_{2}-K_{a}\right)}e^{-K_{a}t}\;+\frac{{[K_{a1}\left(K_{21}-\lambda_{1}\right)+(K}_{21}K_{a2})]}{\left(K_{a}-\lambda_{1}\right)\left(\lambda_{2}-\lambda_{1}\right)}e^{-\lambda_{1}t}\;+\frac{{[K_{a1}\left(K_{21}-\lambda_{2}\right)+(K}_{21}K_{a2})]}{\left(K_{a}-\lambda_{2}\right)\left(\lambda_{1}-\lambda_{2}\right)}e^{-\lambda_{2}t}]$$

or:

$$C_{1}e^{-K_{a}t}+C_{2}e^{-\lambda_{1}t}+C_{3}e^{-\lambda_{2}t}\;\;\;\;\left(K_{a}\;>\;\lambda_{1}\;>\;\lambda_{2}\right)$$

Calculation of the absorption rate constants, *K*_a1_ and *K*_a2_:

(16) $$\frac{C_{2}}{C_{3}}=\frac{{[{(K}_{a}-K_{a2})\left(K_{21}-\lambda_{1}\right)+(K}_{21}K_{a2})]\left(K_{a}-\lambda_{2}\right)\left(\lambda_{1}-\lambda_{2}\right)}{{[{(K}_{a}-K_{a2})\left(K_{21}-\lambda_{2}\right)+(K}_{21}K_{a2})]\left(K_{a}-\lambda_{1}\right)\left(\lambda_{2}-\lambda_{1}\right)}$$

(17) $$N=\frac{\left(K_{a}-\lambda_{2}\right)\left(\lambda_{1}-\lambda_{2}\right)}{\left(K_{a}-\lambda_{1}\right)\left(\lambda_{2}-\lambda_{1}\right)}=\frac{\left(K_{a}-\lambda_{2}\right)}{\left(\lambda_{1-}K_{a}\right)}$$

Then:

(18) $$\frac{C_{2}}{{N⸳C}_{3}}=\frac{K_{a}\left(K_{21}-\lambda_{1}\right)+K_{a2}\lambda_{1}}{K_{a}\left(K_{21}-\lambda_{2}\right)+K_{a2}\lambda_{2}}$$

and after reorganizing:

(19) $$K_{a2}=\frac{\frac{C_{2}}{{N⸳C}_{3}}K_{a}\left(K_{21}-\lambda_{2}\right)-K_{a}\left(K_{21}-\lambda_{1}\right)}{\lambda_{1}-\frac{A_{2}}{{N⸳A}_{3}}\lambda_{2}}$$

when $K_{21}$ is derived after dividing $\lambda_{1}⸳\lambda_{2}$ by $K_{10}$. ($K_{10}$ is obtained from the pharmacokinetic profile following IV bolus administration of the drug).

(20) $$K_{a1}{=K}_{a}-K_{a2}$$

## 2. Results and Discussion

Cannabidiol is a highly lipophilic compound with a low absorption rate when administered orally. When CBD was co-administered orally with chocolate cookies, its bioavailability was only 6% [22]. On the other hand, the full bioavailability provided by the invasive IV administration of hydroalcoholic CBD solutions for injection cannot naturally be medically practiced; however, it is definitely required for pharmacokinetic evaluation of CBD and other cannabinoids. The intranasal administration of CBD, which is non-invasive and compliant, may be a good alternative to the oral administration if an appropriate formulation is applied. In particular, the formulation of nano-sized particles is one of the best alternatives for brain targeting via the nasal route. Cannabidiol pharmacokinetics was evaluated after intranasal administration of CBD in nanoparticles to rats, while each animal was used to represent a single timepoint for plasma and brain level. Doses of 100 µg of CBD (0.39 ± 0.04 mg/kg body weight), loaded in 5% divanillin-crosslinked starch-based nanoparticles (SNPs), were intranasally administered to the rats (20 μL volumes/nostril × 2). Table 1 presents the properties of CBD-SNPs used in this study.

The intranasal administration of CBD to the animals was compared to the intravenous injection of 100 µg doses of CBD in solution (0.36 ± 0.01 mg/kg body weight). A group of 6–12 animals for each time point per each route of administration was used. When a drug is administered intravenously, it is first distributed in the plasma and from there to the various organs and tissues. These organs and tissues can be classified as well-perfused (kidney, liver) and poorly perfused (skeletal muscle, fat). Two compartmental pharmacokinetic models usually analyze combined plasma and the well-perfused organs into one single compartment (the central compartment), from which elimination occurs, especially for active agents with high hepatic clearance, such as CBD. The second compartment represents the body organs and tissues, to which the drug is distributed in the course of time. When a drug is given via extravascular administration and the absorption process is involved, such as via the commonly used oral route, the distribution to the second compartment is usually masked and invisible. However, since intranasal administration is unique by involving two absorption processes, a two-compartmental model can be considered. The basis of the intranasal model (which is described in the Theoretical Section) is that the drug splits into two absorption sites, generating two compartments; one portion goes directly to the brain, and the other portion enters to the blood and to the rest of the body. Thus, two first-order absorption processes are considered (absorption rate constants, K_a1_ and K_a2_).

**Figure 2:**
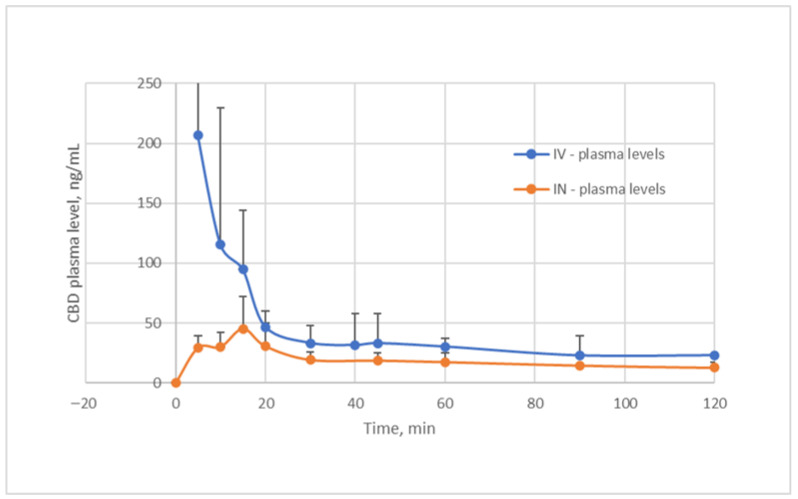
Pharmacokinetic profiles of CBD plasma concentrations after IV (0.36 ± 0.01 mg/kg body weight) and IN (0.39 ± 0.04 mg/kg body weight) administrations.

The pharmacokinetic parameters and profiles of intravenous vs. intranasal cannabidiol administration to rats are presented in Table 2 and Figure 2. The analysis of this drug kinetics revealed: (*a*) plasma CBD concentration–time data after the IV route of administration were best fit by the two-compartmental model, and (*b*) plasma CBD levels and the AUC values obtained after intranasal administration were significantly lower than those recorded after IV administration of comparable CBD doses (ANOVA, *p* < 0.05). The AUC obtained from the mean plasma CBD levels after the IV bolus injection was 13,185.3 ng⸳min⸳ml^−1^, while that obtained from the mean plasma levels following intranasal administration was 6844.3 ng⸳min⸳ml^−1^. The extent of absorption (bioavailability F), which is the ratio of intranasal and intravenous AUCs ([AUC_IN_⸳D_IV_]/[AUC_IV_⸳D_IN_]), was 47.9% (Table 2). Thus, it is evident that there was significantly less bioavailable CBD in the central compartment following intranasal administration; however, the bioavailability is notably higher compared to the oral CBD. Paudel et al. [17] examined the bioavailability of a 200 µg/kg CBD dose in rats after intranasal and intravenous administrations of CBD solution in PEG 400, and found 34–46% bioavailability, which was not improved by enhancers. Although CBD administered via the intranasal route may be lost in part by enzymatic degradation in the nasal mucosa, it bypasses the first-pass effect that is characteristic of drugs taken orally. Yau et al. [5] have already indicated that “intranasal and inhalation drug delivery, being non-invasive and able to achieve fast absorption and increase bioavailability, are attracting increasing interest for CBD applications”.

The elimination rate constant, K_10_, was derived from IV data (Table 2) as follows:

$$K_{21(iv)}=\frac{{C_{2}\cdot \lambda }_{2}{+\;C_{3}\cdot \lambda }_{1}}{C_{2}+C_{3}}$$

$$K_{10}=\frac{\lambda_{1}\cdot \lambda_{2}}{K_{21(iv)}}$$

K_10_ was used for calculating the intranasal-related K_12_ and K_21_ according to the model presented in Figure 1:

$$intranasal\;K_{21}=\frac{\lambda_{1}\cdot \lambda_{2}}{K_{10}}$$

$${intranasal\;K}_{12}=\lambda_{1}+\lambda_{2}-K_{21}-K_{10}$$

**Figure 3:**
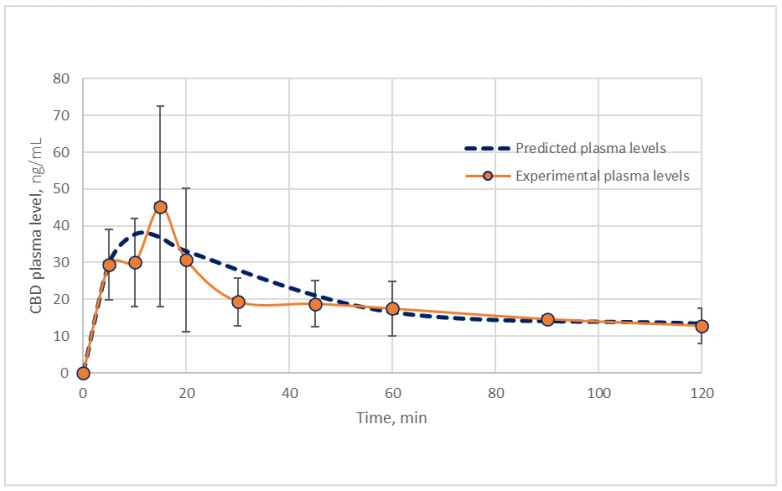
Pharmacokinetic profile of CBD plasma concentrations after intranasal (0.39 ± 0.04 mg/kg body weight) administration, presented as the mean (±SD) of CBD data derived from analyzed plasma samples (Experimental plasma levels) and the predicted data based on Equation (15).

Intranasal-related K_12_ and K_21_ (CBD disposition rate into and from the brain compartment) were found to be 0.0697 min^−1^ and 0.0107 min^−1^, respectively (Table 2). The absorption rate constants, K_a1_ and K_a2_ (Figure 1), were calculated according to Equations (16)–(20) (Theoretical Section). The absorption rate constants following intranasal administration of CBD were 0.0489 min^−1^ representing systemic absorption and 0.623 min^−1^ representing direct brain entry, which accounted for 44% and 56% of the total drug absorption (*F*∙D), respectively (Table 2). By substitution of the intranasal parameters in Equation 15, the pharmacokinetic profile of CBD can be extrapolated for every dose applied (Figure 3), provided that a linear pharmacokinetics exists.

There are a few publications which explored intranasal administrations of CBD. A rapid CBD appearance in the plasma following IN administration was reported, due to prompt absorption through the highly vascularized nasal epithelium into the blood circulation [5]. CBD was detected as early as 0.5 min after IN administration of CBD solution with a *t*_max_ ≤ 10 min [17], which is in agreement with the result presented in this paper, *t*_max_ = 10–15 min. Other publications mentioned that *t*_max_ was approximately 30 min following administration of the CBD solution [15,18] and CBD nano-emulsion [18].

**Figure 4:**
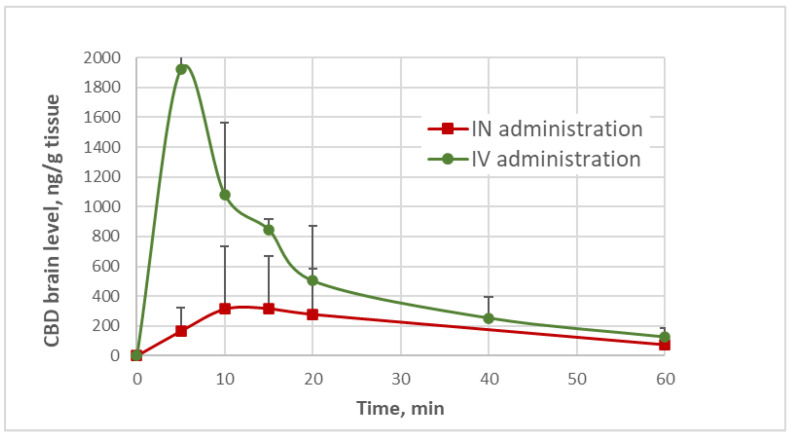
Pharmacokinetic profiles of CBD in the brain after IV (0.36 ± 0.01 mg/kg) and IN (0.39 ± 0.04 mg/kg) administrations. The difference between the mean levels of the two profiles were statistically significant (ANOVA, *p* < 0.05).

**Figure 5:**
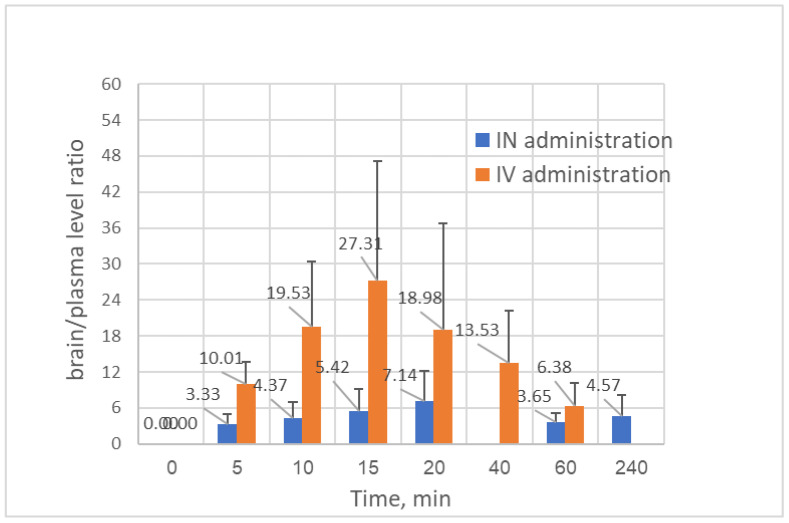
Brain/plasma CBD concentration ratio after IV (0.36 ± 0.01 mg/kg) and IN (0.39 ± 0.04 mg/kg) administrations.

When “nose-to-brain” delivery is considered as a target administration, it is essential to determine brain levels as evidence of effective CNS penetration. The monitoring of brain levels following IN administration of CBD has rarely been available. One recent publication reported a *brain t*_max_ of 2 h following IN administration of CBD solution, and a *brain t*_max_ of 30 min after IN administration of CBD nano-emulsion [18], implying a formulation-dependent drug delivery. Unlike these reports, intranasal administration of CBD-loaded SNPs, which are described in the present paper, showed a *brain t*_max_ of 10 min (Figure 4). The *brain* pharmacokinetic profiles of CBD after IN and IV administrations were obtained after analysis of the organs removed from the animals at predetermined timepoints along with the plasma sampling; thus, each animal was used to represent a single timepoint for plasma and brain level, as described previously. The pharmacokinetic profiles in the brain organ following the two administrations are presented in Figure 4, demonstrating relatively rapid input of CBD to the brain, with a *t*_max_ of 5 min after IV bolus injection of CBD solution, and a *t*_max_ of 10 min after IN administration of a comparable dose of CBD in the SNP carriers. Table 3 summarizes the pharmacokinetic parameters of CBD quantities monitored versus time in the brain compartment, demonstrating a brain bioavailability of 28.5% by the nasal route (compared to the brain bioavailability after IV bolus of CBD solution). However, since only about half of the CBD dose (F = 47.9%) was absorbed into the body (calculated according to the plasma data), 59.5% of the CBD intranasal dose entered into the brain by both direct delivery and blood circulation, which is just another way of calculating the “drug targeting efficiency”. This number resembles the common expression of drug targeting efficiency (DTE% = 59.4%; Equation (22) in Section 3), which reflects the brain-to-plasma partitioning ratio when intranasal administration is compared to IV injection. The obtained DTE% value (59.4%) may also correspond to the “absorption rate constant” obtained for CBD intranasal administration, K_a2_ = 0.623 min^−1^, representing a direct brain entry of 56% from the total drug absorption (Table 2). Figure 5 demonstrates another mode of evaluating the “nose-to-brain” efficacy, which is simply the brain-to-plasma CBD concentration ratio. After CBD had been intravenously administered, relatively high brain/plasma ratios (maximal average of 27.3 after 15 min from injection) were measured in all sampling time points, obviously due to easy and unrestricted BBB passage of the compound. After intranasal administration, the brain/plasma ratios were between 3.3 and 7.1 (the maximal average was 7.1 after 20 min from the administration), highlighting the beneficial delivery of CBD by nanoparticles.

Considering the interesting finding that the half-lives of CBD elimination (*t*_1/2 λ2_ = 20.1 and 17.9 min for IV and IN, respectively) were quite similar for both modes of administration, it is then obvious that the relatively lower absolute bioavailability of IN administration is associated with a difference in the rates of CBD entry into the brain. The input rate constant of CBD to the brain after IV administration (K*_in_* = *brain* K_12(IV)_) was 3.087 min^−1^ (or t_1/2(in)_ = 0.22 min), indicating that CBD gains a fast and easy passage through the blood–brain barrier (BBB). In contrast, the input rate constant of CBD to the brain after IN administration (K*_in_* = K_a2_ + *brain* K_12(IN)_) was 0.0976 min^−1^ (or t_1/2(*in*)_ = 7.1 min), which linked with the slower CBD absorption phase to the CNS (t_1/2 λ1 (IN)_ = ln2/0.079 = 8.75 min vs. t_1/2 λ1 (IV)_ = ln2/0.185 = 3.75 min). Another factor that can express the input extent of drugs in the peripheral (whole body disposition) compartment is the output–input exchange ratio (R). Thus,

for IV administration:

$$R_{IV}=\frac{K_{12(IV)}+K_{10}}{K_{21(IV)}}=8.03$$

For IN administration:

$$R_{IN}=\frac{K_{12(IN)}+K_{10}}{K_{a1}+K_{21(IN)}}=1.63$$

These values show that the overall peripheral organs and tissues have a relatively higher exchange rate if CBD is delivered directly to the blood vessels by IV injection. However, using the specific two-compartmental model in which the brain is the outer compartment, there is a lower exchange rate to the brain if CBD is absorbed through the nasal route.

## 3. Materials and Methods

### 3.1. Materials

Maize (corn) starch was obtained from Hopkin & Williams Ltd. (Chadwell Heath, Essex, UK). Divanillin (DV) was prepared by an enzymatic reaction of vanillin (Sigma-Aldrich Israel Ltd., Rehovot, Israel) in the presence of horseradish peroxidase and hydrogen peroxide. Mannitol was obtained from Sigma-Aldrich (Saint Louis, MO, USA), and urea from E. Merck (Darmstadt, Germany). Sodium hydroxide AR, hydrochloric acid 32% AR, and ethyl alcohol AR were purchased from BioLab Ltd. (Jerusalem, Israel). High-performance liquid chromatography (HPLC)-grade solvents were obtained from J.T. Baker (Mallinckrodt Baker, Inc., Phillipsburg, NJ, USA).

### 3.2. Preparation of Starch Nanoparticles

The process of nanoparticles preparation using the “non-solvent dropping nanoprecipitation” technique was previously reported [20]. Briefly, an aliquot of starch slurry adjusted to pH = 3 was diluted with purified water, and divanillin suspension was added. CBD-containing ethanolic solution (used as the non-solvent) was dropwise added to the starch mixture while it was continuously stirred by a magnetic bar. At the completion of the nanoprecipitation process, the remaining ethanol was evaporated using a rotary evaporator, and the starch nanoparticles dispersion was lyophilized with mannitol (0.1% *w*/*v*). CBD-loaded SNP powder was kept refrigerated in closed containers until use.

### 3.3. Determination of CBD Content in SNPs

Accurately weighed (3–4 mg) lyophilized CBD-loaded SNP powder was dispersed in water/methanol (1:1), sonicated for 8 min, and centrifuged at 17,000 *rcf* for 12 min. The supernatant was collected (800 µL) and analyzed by HPLC. The extract was injected into an HPLC system (1260 Infinity II, Agilent Technologies Inc., Santa Clara, CA, USA), equipped with a prepacked column (BetaSil C18, 5 μm, 250 mm × 4.6 mm, Thermo Fisher Scientific, Waltham, MA, USA). The samples were chromatographed using a mobile phase consisting of acetonitrile/acetic acid 0.1% (80:20) at a flow rate of 1 mL/min. Calibration curves, peak areas measured at 208 nm for CBD versus drug concentrations, were constructed by running standard drug solutions in methanol for each series of chromatographed samples. The limit of detection (LOD) was 0.01 µg/mL and the limit of quantification (LOQ) was 0.05 µg/mL. 

Entrapment efficiency percentage (EE%) was calculated according to the following equation:

(21) $$\mathrm{EE}\%\;=\;\frac{Mass\;of\;CBD\;in\;formulation}{Total\;mass\;of\;CBD\;used\;in\;formulation}\;\times \;100$$

CBD loading in the nanoparticles was expressed as the mass of CBD entrapped in the formulation per 1 g of starch.

### 3.4. Nanoparticle Tracking Analysis (NTA)

Measurements were performed using a NanoSight NS300 instrument (Malvern Instruments Ltd., Malvern, UK), equipped with a 642 nm red laser module and 450 nm long-pass filter, and a camera operating at 25 frames per second, capturing a video file of the particles moving at a constant flow rate from a syringe on a syringe pump. The software for capturing and analyzing the data (NTA 3.4) calculated the hydrodynamic diameters of the particles by using the Stokes–Einstein equation.

### 3.5. Animals

All animal treatments followed protocols reviewed and approved by the Institutional and Use Committee, Ben-Gurion University of the Negev, which complies with the Israeli Law of Human Care and Use of Laboratory Animals. Sprague–Dawley rats (male, 250–300 g body weight, Harlan Laboratories, Ein Karem, Jerusalem, Israel) were used in this study. All animals were housed in polycarbonate cages and maintained on a 12/12 h light/dark cycle under controlled temperature and humidity conditions. The rats had ad libitum access to food and water.

### 3.6. Intranasal Administration (IN)

Animals were randomly divided into groups according to the length of the post-administration period. The dose administered to each animal’s nostrils was 100 µg CBD in nanoparticles, which accounted for 0.39 ± 0.04 mg/kg body weight. SNP powder was suspended in water immediately before nasal installation. The applied volume was 20 μL/nostril (total of 40 μL containing 100 µg CBD). The animals were sedated with isoflurane vapor to facilitate the administration. After a predetermined period from administration (i.e., 5, 10, 15 min, etc.) the animals were euthanized by CO_2_ aspiration. Then, a blood sample was taken by cardiopuncture into heparinized tubes (30 units of heparin per tube). Blood in heparinized tubes was centrifuged at 15,000 *rcf* for 15 min and the separated plasma was transferred into vials and kept at −20 °C until sample preparation. The brain was carefully removed, frozen at −80 °C, lyophilized for 24 h and kept at −20 °C until sample preparation.

### 3.7. Intravenous Administration (IV)

As in IN administration, animals were randomly divided into groups according to the length of the post-administration period. The animals were sedated by an intraperitoneal injection of Ketamine (75 mg/kg) and Xylazine (5 mg/kg). The IV solution was injected into the tail vein using a 27 G needle. The dose used for IV administration was identical to the IN dose, i.e., 100 µg CBD per animal, which accounted for 0.36 ± 0.01 (IV) mg per kg of body weight. The CBD solution for injection was prepared by dissolving 10 mg of CBD powder in 500 µL of dehydrated ethanol, followed by an addition of 75 µL of Tween-80 and 9425 µL of 0.9% sodium chloride solution. The final solution contained 100 µg/0.1 mL with 5% ethanol and 0.75% Tween-80. The solution was freshly prepared for each injection session. All animals were treated identically to the procedures described in Section 3.6 regarding euthanasia, blood sampling and brain extraction.

### 3.8. Plasma and Brain Samples Preparation

Frozen plasma samples were thawed at room temperature. Into 200 µL of a plasma sample, 50 µL of methanolic internal standard (ISTD) CBD-D_3_ solution (40 µg/mL) and 350 µL of acetonitrile were added, then the combined solution was vortexed and centrifuged at 15,000 *rcf* for 15 min, and the separated supernatant was immediately analyzed by liquid chromatography–tandem mass spectrometry (LC-MS/MS). CBD standards were prepared using 200 µL of plasma (taken from an untreated animal), 100 µL of standard CBD solution (methanolic CBD solution), 50 µL of ISTD CBD-D_3_ solution (40 µg/mL) and 250 µL of acetonitrile using the same procedure as described above. The dried brain tissues were ground just before analysis, 0.4 mL of ISTD CBD-D_3_ solution (40 µg/mL) and 1.6 mL of methanol were added, and the mixture was centrifuged at 3351 *rcf* for 10 min at 25 °C. The supernatant (≈1.2 mL) was separated. Then, 250 µL of this supernatant was added to 350 µL of acetonitrile. The mixture was centrifuged at 15,000 *rcf* for 15 min and the separated supernatant was analyzed by LC-MS/MS.

### 3.9. Quantification of CBD by Liquid Chromatography–Tandem Mass Spectrometry (LC-MS/MS)

LC was performed on the Shimadzu LC system (UFLC series, Shimadzu Corp., Kyoto, Japan), with a ReproSil-Pur C18 column (100 mm 2 mm, 5 μm, Dr. Maisch, Ammerbuch, Germany). The mobile phase was acetonitrile with 0.2% formic acid. The flow rate was 0.2 mL/min, and the injection volume was 5 µL. Both the column and the autosampler were kept at room temperature. Tandem mass spectrometry was performed using a SCIEX API 3200 triple quadrupole mass spectrometer (SCIEX, Toronto, ON, Canada) equipped with an electrospray ionization (ESI) source operating in a positive ion mode. Quantification was carried out in multiple reaction monitoring (MRM) mode using the transitions *m*/*z* 315.2 → 193.2 for CBD and *m*/*z* 318.2 → 196.2 for CBD-D_3_ (retention time: 4.0–4.5 min for both). The collision energy was set to 30 eV. The ion spray voltage was 5000 V, the source temperature was 450 °C, and the curtain gas was 40 psi. The ion source gas 1 (GS1) and the ion source gas 2 (GS2) were both set to 20 psi. Nitrogen was used for both the collision gas (CAD = 5) and the curtain gas. Repeated calibration curves, peak areas measured for CBD versus drug concentrations, were constructed by running freshly prepared CBD methanolic solutions or CBD-spiked plasma extracts (including internal standards) for each series of chromatographed samples. The limit of detection (LOD) was 0.2 ng/mL and the limit of quantification (LOQ) was 1 ng/mL.

### 3.10. Pharmacokinetic Analysis

A PKSolver Add-In software (Microsoft Excel for Microsoft 365 MSO Version 2602) [23] containing a compartmental pharmacokinetic analysis (CA) was used to calculate the pharmacokinetic parameters. The absolute bioavailability was calculated by the plasma AUC_IN_/AUC_IV_ ratio. Drug targeting efficiency (DTE) was calculated as follows:

(22) $$DTE\%=\frac{{\left({AUC}_{brain}/{AUC}_{plasma}\right)}_{IN}}{{\left({AUC}_{brain}/{AUC}_{plasma}\right)}_{IV}}\times 100$$

### 3.11. Statistical Analysis

The kinetic studies were conducted by using at least six animals for each time point and the drug levels are represented as average ± SD. The unweighted means analysis of variance (ANOVA) test for the differences among group means was used and differences at a *p* < 0.05 were considered significant.

## 4. Conclusions

The global CBD market has been experiencing substantial growth alongside a large number of research publications. The major problems reflected in previous publications were the extremely low and variable oral bioavailability due to a first-pass metabolism, and hepatotoxicity seen in patients taking high doses. The development of low-dose nasal formulations would be beneficial for the delivery of sufficient CBD to the brain through rapid absorption and improving the patient’s safety. In the current study, we demonstrate the importance of pharmacokinetic evaluation in the development of nasal delivery systems to the brain. The present pharmacokinetic study showed that the absolute bioavailability of CBD after intranasal administration was relatively high, 47.9%, followed by a significantly lower systemic exposure compared to IV injection of a similar dose. We have proposed a pharmacokinetic model which applicably describes and predicts the disposition pathways following intranasal administration, and especially those nasal delivery systems designated for brain targeting. By using this new model, it was revealed that 44% of the absorbed CBD was delivered after intranasal administration into the systemic circulation, while 56% was trafficked directly into the brain. The maximal mean brain/plasma ratio was 7.1 after 20 min from nasal administration (B/P > 1), highlighting the beneficial delivery of CBD by the starch-based nanoparticles. However, this obtained value was significantly lower than the value of the brain/plasma ratio obtained after IV administration. Future development work of intranasal CBD and intranasal cannabinoids in general should focus on the discovery and development of nano-formulations that improve transport efficiency to the brain while reducing the dosage to minimum.

## Figures and Tables

**Table 1:** Properties of cannabidiol-loaded starch nanoparticles (CBD-SNPs), which were introduced to the pharmacokinetic study by intranasal administration.

CBD Loading in SNPs	64.5 ± 10.8 mg/g Starch
Entrapment efficiency (EE%)	25.7 ± 4.2%
Mean particle size (±SD)	241.1 ± 68.2 nm
Polydispersity Index (PDI ± SD)	0.17 ± 0.01
SNP concentration (±SD)	2.14 × 10^10^ ± 8.6 × 10^9^ particles per 1 mg of lyophilized powder

**Table 2:** Summary of the pharmacokinetic parameters of cannabidiol following intravenous (IV) administration of a solution, and intranasal (IN) administration of starch-based nanoparticles to rats as monitored in plasma. K_a1_ and K_a2_ are the absorption rate constants of the drug after IN administration, K_10_ is the elimination rate constant, and K_12_, K_21_ and K_12(IN)_, K_21(IN)_ are the distribution rate constants of the drug after IV and IN administration, respectively.

	IV *Plasma*	IN *Plasma*
**Dose, mg/kg**	0.36 ± 0.01	0.39 ± 0.04
**C1**	---	1259.3
**C2**	329.0	1200.3
**C3**	33.3	18.0
**λ_1_, min^−1^**	0.1286	0.1051
**λ_2_, min^−1^**	0.0031	0.0028
***t*_1/2_ λ_2_, min**	222.8	245.9
**K_a,_ min^−1^**	---	0.1112
***t*_1/2 Ka_, min**	---	6.23
**V_1_, mL**	276	---
**AUC_0–∞_, ng** **⸳** **min/mL**	13,185.3	6844.3
***F*** **, %**	100	**47.9**
**K_10_, min^−1^**	**0.0275**	---
**K_12_, min^−1^**	0.0897	---
**K_21_, min^−1^**	0.0146	---
**K_12(IN)_, min^−1^**	---	0.0697
**K_21(IN)_, min^−1^**	---	0.0107
**K_a1,_ min^−1^**	---	0.0489 (**44%**)
**K_a2,_ min^−1^**	---	0.0623 (**56%**)

**Table 3:** Summary of the brain pharmacokinetic parameters of cannabidiol following intravenous (IV) administration of a solution and intranasal (IN) administration of starch-based nanoparticles to rats as monitored in plasma.

	IV *Brain*	IN *Brain*
**Dose, mg/kg**	0.36 ± 0.01	0.39 ± 0.04
**λ_1_, min^−1^**	0.1850	0.0792
**λ_2_, min^−1^**	0.0344	0.0387
***t*_1/2_ λ_2_, min**	20.1	17.9
**K*_in_*** *_,_ min^−1^	3.087	0.0976
***t*_1/2(*in*)_, min**	0.22	7.10
**AUC_0–∞_, ng⸳min** **	14,700.1	4536.1
**F** *_brain_* **, %**	100	**28.5**

Notes: * K***_in_*** is the rate constant of drug entry into the brain. ** AUC was calculated from CBD quantity found in the brain versus time profile.

## Data Availability

The original contributions presented in this study are included in the article. Further inquiries can be directed to the corresponding authors.

## Abbreviations

The following abbreviations are used in this manuscript:

AUC	Area Under the Curve
BBB	Blood–brain barrier
CBD	Cannabidiol
*C*_max_	Maximum concentration
CNS	Central nervous system
DTE	Drug targeting efficiency
DV	Divanillin
EE%	Entrapment efficiency percentage
ESI	Electrospray ionization
HPLC	High-performance liquid chromatography
IN	Intranasal
IV	Intravenous
LC-MS/MS	Liquid chromatography–tandem mass spectrometry
LOD	Limit of detection
LOQ	Limit of quantification
MRM	Multiple reaction monitoring
NPs	Nanoparticles
NTA	Nanoparticle tracking analysis
PEG	Polyethylene Glycol
rcf	Relative Centrifugal Force
SNPs	Starch nanoparticles
*t*_max_	Time of the maximum concentration
W/O	Water-in-Oil
W/V	Weight per Volume
